# Anxiety disorders in acute central nervous system infections

**DOI:** 10.1186/s13052-020-0788-6

**Published:** 2020-02-17

**Authors:** Elena Bozzola, Giulia Spina, Paola Bergonzini, Mauro Bozzola, Massimiliano Raponi, Alberto Villani

**Affiliations:** 1 Pediatric and Infectious Diseases Unit, Bambino Gesù Children Hospital, Rome, Italy; 2 Neuropsychiatric Unit, Bambino Gesù Children Hospital, Rome, Italy; 3 University of Pavia, Pavia, Italy; 4 Sanitary Direction, Bambino Gesù Children Hospital, Rome, Italy

**Keywords:** Acute central nervous system infections, Neuropsychological disorders, Children

## Abstract

**Background:**

Patients affected by acute central nervous system (ACNS) infectionsmay present different complications, including neuropsychological disorders. Nevertheless, psychopathological impairment has been rarely measured by appropriate and validated tests.

**Material and methods:**

Survivors of childhood ACNS infections admitted to the Bambino Gesù Children’s Hospital, Rome, Italy, from June 2013 to June 2015 were re-evaluated at follow-up from June 2016 to June 2017. Both patients and their parents underwent a psychological interview and neuropsychological tests (the Leiter International Performance Scale – revised (Leiter-R), the Child Behaviour Checklist (CBCL), the K-SADS-PL test).

**Results:**

Thirty children were included in the study. The mean score of IQ and fluid reasoning was within the normal range. A percentage of 20% of the children enrolled showed criteria for generalized anxiety disorder.

**Conclusion:**

Our study revealed the importance of follow-up evaluations after ACNS infections, in order to prevent mayor psychological sequelae and to perform treatment or rehabilitation.

## Background

Acute central nervous system (ACNS) infections such as encephalitis, meningitis, and cerebellitis are related to considerable rates of morbidity and mortality despite appropriate therapy.

Psychopathological impairment as a complication of ACNS infection has been described in literature, but rarely measured by appropriate and validated tests [1–3].

In details, neurocognitive, educational, and psychological difficulties may affect meningitis survivors during childhood and early adolescence. Also in apparently healthy patients, depressive and anxiety symptoms, psychological and behavioural problems, and increased risk of psychotic experiences have been reported [4].

In a previous study, we investigated if psychopathological problems may affect ACNS infections survivors and if we can early detach them. At the 1 year follow-up, anxiety problems such as dependency, fears, worries, nervousness, developed in quite half of patients. Moreover, somatic problems, like aches, headaches, nausea, vomiting, had been detached in quite a third of patients. Finally, “affective problems” such as crying, self-harming, worthlessness, guilt, tiredness, sadness, were reported in 29% of patients [5].

### Aim of the study

The current study aims to investigate whether children previously affected by ACNS infection develop any neuropsychological impairment measured with validated tests, during a longer follow-up observation.

## Patients and methods

Participants were a consecutive sample of survivors of childhood ACNS infections, admitted to the Bambino Gesù Children’s Hospital, Rome, Italy, from June 2013 to June 2015 and then re-evaluated at follow-up from June 2016 to June 2017. The inclusion criteria was an age older than 3 years at follow-up.

The exclusion criteria were immunodeficiency, malignancy or underlying psychiatric pathology. The eligible population was divided into four groups according to the diagnosis. Group A included meningitis, Group B cerebellitis, Group C encephalitis, Group D meningoencephalitis.

Acute cerebellitis was defined in the presence of ataxia, unsteady gait or fine motor movement, trembling of the head and trunk in an upright position and the extremities when attempting to move against gravity [6].

For the diagnosis of meningitis, suspected on clinical symptoms, the analysis of the cerebrospinal fluid was required [7].

According to literature, a patient has been defined as affected by meningoencephalitis in case of meningitis associated to an altered level of consciousness and/or focal neurological signs, associated to pathological change of the electroencephalogram study or neuroimaging findings [7].

Encephalitis is characterized by a typical clinical pattern with a brief ‘flu-like’ prodrome followed by severe headaches, vomiting and altered consciousness. Patients affected by encephalitis may also have meningism, seizures and focal neurological signs [8].

Both patients and their parents underwent a psychological interview during a follow-up control.

The tests were performed by a psychologist and were selected according both to patient’s age and to the ability to collaborate with the doctor. Potential participants were invited to take part to the study by returning signed consent forms.

### Neuropsychological tests

The following tests were performed to patients: the Leiter International Performance Scale – revised (Leiter-R), the Child Behaviour Checklist (CBCL), the K-SADS-PL test.

#### Cognitive profile

The Leiter-R scale, developed as a nonverbal intelligence measurement tool, may be used to assess children, adolescents, and young adults aged from 2 years, 2 months to 20 years, 11 months, who could not be reliably and validly assessed with traditional intelligence tests. In details, the test is manageable, and does not require proficiency in perceiving, manipulating, and reasoning with words or numbers, or using any other materials traditionally identified as “verbal”. All administration instructions are adapted to a nonverbal format. Because of these features, this scale is widely utilized to assess the intellectual function of children who cannot be tested with standard intelligence tests [2].

#### Psychopathological evaluation

The CBCL is a parent/ caregiver report form to screen for emotional, behavioural, and social problems, such as anxiety, depression, aggressive behaviour, etc [3]. The CBCL also has a scale scores associated with disorders from the Diagnostic and Statistical Manual of Mental Disorders (DSM-IV-TR; American Psychiatric Association 2000): anxiety, oppositional defiant disorder, conduct problems, somatic problems, affective problems and attention deficit disorder.

There are two versions of the checklist: the preschool checklist (CBCL/11/2–5) used for children from 18 months to 5 years old; the school-age version (CBCL/6–18) is for children aged 6 to 18 years [9, 10].

#### Interview

The *K-SADS-PL test* is a diagnostic interview administered by psychologists to both children aged 6–11 years and their parents. It is used for the evaluation of psychopathological disorders.

The theoretical model of Reference is essentially made up of the DSM-III-R and DSM-IV. The final score results from the total of all the data collected from various sources available (family, children, teachers, paediatricians, etc.) [4]. In particular, it is composed of six items: an unstructured interview, a diagnostic screening interview that analyzes primary symptoms referred to the diagnosis established using the test, an additional checklist, diagnostic supplements, a description of patient’s medical history, a scale for overall assessment of the child’s current functioning [4].

#### Statistical analysis

A basic statistical descriptive analysis was performed to analyze the sample.

The statistical analysis includes a descriptive analysis of each variable. Quantitative results are expressed as the mean and standard deviation (SD). The qualitative results are expressed in absolute and relative (percentage) values. Subsequently, a bivariate analysis was performed to study the influence of the different clinical and epidemiological variables collected on the dependent variables.

The confidence intervals were 95%, and the statistical package used was Statistica Release 7.

## Results

During the period study, 84 children were admitted for ACNS infections to our hospital. From June 2016 to June 2017, 30 of these children were included in the study as they met all the inclusion criteria. Among them, 18 were included in group A, 7 in group B, 1 in group C and 4 in group D.

The other 54 were excluded as they were younger than 3 years.

No physical sequelae were reported among enrolled patient.

The mean time from ACNS hospitalization to neuropsychological evaluation was 6 years; the mean age of children during the neuropsychological evaluation was 8.6 years (range from 3.08 to 15.83 years). Gender distribution was balanced (12 females and 18 males).

Table 1 summarizes the demographic data of patients. Table 2 concerns the bivariate analysis on the clinical and the epidemiological variables.

**Table 1:** Demographic data of the patients

	Meningitis	Cerebellitis	Encephalitis	Meningoencephalitis
Number of patients (n, %)	18, 60%	7, 23%	4, 13%	1, 3%
Age, years (mean)	8,6 years	8,1 years	9,4 years	5,9 years
Age, years to admission (n)	10 months	4,2 years	3,4 years	2 months
Sex male (n, %)	9, 30%	6, 20%	2, 7%	0
Race Caucasian (n, %)	18, 60%	6, 20%	4, 13%	1, 3%

**Table 2:** Bivariate analysis on clinical and epidemiological variables

Age (years)	3–6	7–10	11–15	TOT
Gender				
Male	8	6	4	**18**
Female	4	4	4	**12**
TOT	**12**	**10**	**8**	**30**

Chi-square statistic is 0.5556; *p*-value is .757465. The result is *not* significant at *p* < .05

Leiter-R scale was administered to 30 children (aged 3–15 years) to assess nonverbal cognitive functioning. Parents/guardians completed the CBCL and participated to K-SADS interview to assess emotional /behavioural issues.

The mean score of IQ and fluid reasoning was within the normal range of mind (IQ mean 97.82, SD 17.78; Fluid Reasoning mean 95.62, SD 16.49).

The average results obtained in the CBCL’s internalizing and externalizing area were not included in clinical significance range. In particular, internalizing problem T score mean observed was 49.2 while externalizing problem T score mean was 42.66.

A percentage of 20% of the children enrolled showed criteria for generalized anxiety disorder during the semi-structured K-SADS interview. In particular, six patients (3 males and 3 females) obtained a diagnosis of anxiety disorder during the evaluation, five patients with a previous diagnosis of meningitis and one child with a previous diagnosis of cerebellitis; only one child obtained attention deficit and hyperactivity disorders diagnosis.

**Fig. 1:**
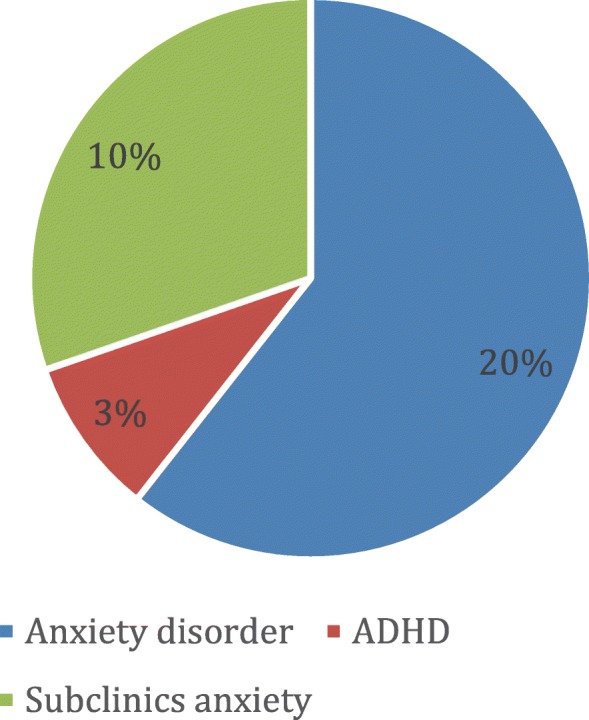
Anxiety disorders resulting from semi-structured K-SADS interviews

Moreover, three patients presented few characteristics of anxiety disorder without complete criteria for a psychopathological diagnosis (Fig. 1).

## Discussion

Our study revealed that some patients admitted because of ACNS infections, in particular affected by meningitis, developed anxiety disorders during the follow-up evaluation.

Similarly to our results, Russel Viner et al., showed that children with meningitis were significantly more likely to have anxiety disorder such as conduct disorder and attention-deficit hyperactivity disorder with high probability than controls. In particular, they observed a percentage of 37% of patients having generalised anxiety disorders similarly to our sample (20%) [11]. According to literature, psychopathological impairment may be detached after a few years from the ACNS admission. A percentage of 22% of survivors was diagnosed with significant psychological disorder roughly 3–5 years after disease, as well as in our report [11].

Anxiety and mood disorders are among the most frequent psychopathological impairment and may led to restlessness, being easily fatigued, difficulty concentrating, irritability, muscle tension, and sleep disturbance [12, 13].

Conversely to our results, some studies highlights that children and young people who have survived meningitis are significantly more likely to have a borderline low IQ (< 85), both verbal and non-verbal deficits across all aspects of memory and sometimes in multiple aspects, poorer executive function leading to problems with organisation and planning. In particular, major intellectual disability (IQ < 70) was rare however, about 11% of survivors had either low or borderline IQ (< 85), which in modern society is associated with increased need for educational support and poor educational, vocational, and mental health outcomes in adolescence and adult life [11, 14].

## Conclusion

The linkages between ACNS infections and mental health conditions observed in this study highlight potentially important avenues for disease prevention and control efforts, areas for further research, and potential translational studies aimed at further improving public health (e.g., immunizations), clinical interventions and access to treatment. Moreover, our study revealed the importance of follow-up evaluations after any ACNS infections, in order to prevent mayor psychological sequelae and make strategies to perform the best treatment and rehabilitations programs. In particular, our data, should prove useful in the assessment of ACNS infections survivors to introduce specific psychological tests as routine screening for psychological disorders and cognitive deficits.

A potential limit of the study is the small sample size, correlated to the rarity of the disease. Additional studies are necessary to explain the causal relationship of the ACNS to the psychological sequelae. They will clarify the mechanisms for interactions between mental health and ACNS infections in order to assist patients, reduce morbidity and mortality and improve their quality of life.

## Data Availability

The materials analyzed during the current study are available at Bambino Gesù Children Hospital, Rome at the room of Dr. Bergonzini.
